# Modelling intentions to provide smoking cessation support among mental health professionals in the Netherlands

**DOI:** 10.1186/s12971-016-0096-5

**Published:** 2016-08-26

**Authors:** Matthijs Blankers, Renate Buisman, Petra Hopman, Ronald van Gool, Margriet van Laar

**Affiliations:** 1 Netherlands Expertise Centre on Tobacco Control (NET), Trimbos Institute, Utrecht, The Netherlands; 2 Department of Research, Arkin Mental Health Care, Amsterdam, The Netherlands; 3 Department of Psychiatry, Academic Medical Centre, University of Amsterdam, Amsterdam, The Netherlands; 4 Centre for Child and Family Studies, Leiden University, Leiden, The Netherlands; 5 GGz inGeest Mental Health Institute, Amsterdam, The Netherlands; 6 Cluster of Nursing, Leiden University of Applied Sciences, Leiden, The Netherlands

**Keywords:** Psychiatry, Survey research, Treatment and intervention, Structural equation modelling

## Abstract

**Background:**

Tobacco use prevalence is elevated among people with mental illnesses, leading to elevated rates of premature smoking-related mortality. Opportunities to encourage smoking cessation among them are currently underused by mental health professionals. In this paper, we aim to explore mechanisms to invigorate professionals’ intentions to help patients stop smoking.

**Methods:**

Data stem from a recent staff survey on the provision of smoking cessation support to patients with mental illnesses in the Netherlands. Items and underlying constructs were based on the theory of planned behaviour and literature on habitual behaviour. Data were weighted and only data from staff members with regular patient contact (*n* = 506) were included. Descriptive statistics of the survey items are presented and in a second step using structural equation modelling (SEM), we regressed the latent variables attitudes, subjective norms (SN), perceived behavioural control (PBC), past cessation support behaviour (PB) and current smoking behaviour on intentions to provide support. In optimisation steps, models comprising a subset of this initial model were evaluated.

**Results:**

A sample of 506 mental health workers who had direct contact with patients completed the survey. The majority of them were females (70.0 %), respondents had an average age of 42.5 years (*SD* = 12.0). Seventy-five percent had at least a BSc educational background. Of the respondents, 76 % indicated that patients should be encouraged more to quit smoking. Respondents were supportive to train their direct colleagues to provide cessation support more often (71 %) and also supported the involvement of mental health care facilities in providing cessation support to patients (69 %). The majority of the respondents feels capable to provide cessation support (66 %). Two thirds of the respondents wants to provide support, however only a minority (35 %) intends to actually do so during the coming year. Next, using SEM an acceptable fit was found of the constructs derived from the theory of planned behaviour and literature on habitual behaviour to the weighted data (*χ*^2^ (322) = 1188, *p* < .001; RMSEA = 0.067; CFI = 0.983), after removal of insignificant latent variables (SN and current smoking) and inclusion of covariates. Attitudes, PBC and PB of staff are the strongest identified correlates of intention toward providing cessation support to patients. SN and staff smoking behaviour were found to be weaker, non-significant correlates.

**Conclusions:**

To nudge staff towards providing cessation support to people with mental illnesses one should aim at influencing attitudes and perceived behavioural control.

**Electronic supplementary material:**

The online version of this article (doi:10.1186/s12971-016-0096-5) contains supplementary material, which is available to authorized users.

## Background

Tobacco use prevalence is elevated among people with mental illnesses, compared to the general population. Estimates indicate that on average, smoking prevalence is two to four times higher among people with mental illnesses than in the general population [1]. People with schizophrenia have notably high smoking prevalence rates: a review by de Leon and Diaz [2] estimates the global daily smoking rate for people with schizophrenia to be 62 %. Common mental disorders such as depression (37 %), bipolar disorder (69 %) and substance use disorders (77 %–93 %) also have been found to be associated with high smoking rates [1, 3, 4] compared to smoking prevalence estimates of the World Health Organisation for the general population worldwide (21 % smoked tobacco in 2013 [5]).

As a consequence, premature smoking-related mortality is common among people with mental illnesses. For example, based on data from individuals hospitalized with a primary psychiatric diagnosis in California from 1990 to 2005, mortality was associated with tobacco smoking in 23,620 of the 44,469 patients with schizophrenia (53 %) [1]. In comparison, an estimated 480,000 [6] of the 2,596,993 deaths in the general population of the United States in 2013 (18 %) died as a consequence of cigarette smoking and exposure to tobacco smoke.

Therefore, a moderate proportion of all premature mortality among people with mental illnesses may be prevented if successful measures would be taken to reduce smoking rates among them. To involve this population in smoking cessation treatment, mental health care facilities are a promising setting [4, 7]. However, based on recent evaluations, many opportunities to encourage and support smoking cessation in mental health care institutes are currently not being used [8–10]. This also applies to the Netherlands [11], where the current study was performed.

In a recent study conducted in the Netherlands, it was found that little more than half (54.7 %) of the staff of inpatient facilities had ever helped a patient stop smoking; 24 % had done so in the last year, whereas 35 % intended to provide cessation support to a patient next year [11]. Smoking cessation support in mental health institutes might not be provided more often for a number of reasons. These include tolerant smoking policies and informal norms regarding the acceptability of smoking among staff [4], staff members’ and patients’ opinions that smoking is often a lesser concern for people with mental illnesses [4], or even that smoking is helpful in reducing symptoms of disorders (eg self-medication hypothesis) [12]. Staff members’ own smoking status is also hypothesised to affect the likelihood they will provide cessation support [13]. Other possible reasons may be a lack of training, skills and support for staff to help patients stop smoking, or the limited availability of (effective) interventions to aid smoking cessation [4].

In the current paper, we aim to explore these and other possible mechanisms underlying staff members’ intentions to help patients stop smoking. In this exploration, constructs from the Theory of Planned Behaviour (TPB) [14] are used. The TPB is an established theory to model (health) intentions and behaviour [15–17]. According to the TPB, intentions are the most proximal determinants of behaviour. Intentions in turn are a product of behavioural attitudes (beliefs, feelings and tendencies towards a behaviour), the subjective norm (SN) regarding a behaviour and perceived behavioural control (PBC), which reflects the perception of being able to perform or control a behaviour. In addition, PBC is also hypothesized to have a direct influence on behaviour [14]. The TPB has frequently served as a basis for designing successful smoking cessation and other addiction treatment programs [18]. The number of studies in which the TPB is applied to modelling and changing clinicians’ behaviour is however smaller. A systematic review published in 2007 [19] identified 20 studies in which the TPB (or its predecessor, the theory of reasoned action) has been used in relation to clinicians’ behaviour. The authors conclude that the small number of studies is striking and unfortunate, as the discrepancy between clinicians’ prescribed (based on evidence based treatment guidelines) and actual behaviour implies a need for more research on possible approaches to narrow this gap [19]. Of the 20 studies that were included, two focussed on the provision of smoking cessation interventions by clinicians, neither of the two focussed on mental health care providers. The study by McCarty and colleagues among 397 staff nurses at four hospitals in the United States found that providing cessation advice was related to attitudes toward offering advice and perceived ability to offer advice [20]. The other study, by Puffer and colleagues found that attitudes and PBC were the most important predictors of intention to offer smoking cessation advice in accordance with coronary heart disease guidelines among community practise nurses in England [21].

Our study aims to contribute to this knowledge base and is (to our best knowledge) the first to evaluate the applicability of the TPB in modelling the intention of mental health care treatment staff to provide cessation support to their patients. Providing cessation support can range from single session brief advice to an extensive psychosocial or pharmacological intervention. We will test whether attitudes, subjective norms, PBC, past cessation support behaviour and current smoking behaviour together are significantly associated with intentions to provide future support. We will also test whether a subset of this model, consisting of only the significant paths between these constructs and intention adequately fits the data. This will identify key constructs to address in order to increase the rate at which mental health care staff will provide cessation support.

## Methods

### Data source

Data were obtained from a survey (August – November 2014) on attitudes, norms, smoking policy, perceived behavioural control, intentions and behaviour towards smoking cessation support in mental health institutes in the Netherlands. Survey items were developed by the authors of the study, with input taken from interviews with the target audience (which were part of the general report [11]), from previous studies on mental health care staff opinions on smoking (cessation) and from the TPB literature [14–19]. The survey frame consists of the 57,310 employees [22] of three types of institutes: (a) integrated mental health care institutes, which usually offer both in- and outpatient mental health care and substance abuse treatment (35 institutes), (b) substance abuse treatment centres (9 institutes), and (c) regional institutes for sheltered housing (20 institutes). Together, these institutes comprise the voluntary inpatient mental health facilities for adults in the Netherlands. At times of the study, 64 institutes were represented by the overarching sector organisation of specialist mental health and addiction care providers. Employees working for these 64 institutes were invited to participate in this internet survey.

### Recruitment of participants

Participants were recruited through invitations circulated among staff by the treatment institutes’ newsletters and via the Trimbos Institute (Netherlands institute of mental health and addiction) website. In order to motivate the target audience to participate, three iPads were raffled off.

### Ethics, consent and permissions

All participants provided informed consent before participating in the survey, in line with the Dutch Medical Research Involving Human Subjects Act. Based on previous consultation with the Netherlands’ Central Committee on Research Involving Human Subjects, survey research as performed for this study is exempted from medical ethics approval.

### Measures

*Attitudes* towards their role in providing cessation support to patients were measured with 12 items, answered on a 5-point Likert scale (range: completely disagree-completely agree). An example of an item is: “Patients should be encouraged more often to quit smoking”.

*Subjective norms* regarding smoking and cessation support in the institutes participants worked for were measured with four items, answered on a 5-point Likert scale. Subjective norms are operationalized as perceived smoking policy, which is an injunctive norm. An example of an item is: “The institute I work for enforces a strict smoking policy”.

*Perceived behavioural control* towards providing cessation support to patients is measured with four items, answered on a 5-point Likert scale. An example of an item is: “If I want to, I am able to help a patient quit smoking”.

*Intention* to provide cessation support to patients in the near future is measured with four items. An example of an item is: “Next year, I intend to help at least one patient quit smoking”.

*Past behaviour* regarding providing cessation support to patients was measured with three items. An example of an item is: “In the past year, I have helped at least one patient quit smoking”.

*Respondent’s smoking behaviour*, comprising of smoking status, time until first cigarette after waking up in the morning (if respondent is a daily smoker, otherwise set to 0) and quit intentions (if applicable) was measured with three items.

### Survey weighting

In order to improve the representativeness of the sample, survey weighting was applied. Weights were calculated in order to optimize the representativeness of our sample regarding type of organization, number of inhabitants of the province, gender, age, part time factor and type of function. Survey weights were estimated using raking calibration in R 3.2.1. As a reference value, information regarding the labour market for mental health workers in the three types of organizations was used [22]. Weight bounds were set at 1/6 (lowest possible weight) and 6 (highest possible weight). All analyses in this paper were performed using unweighted data, and corroborated using weights. In the results section, it is indicated whether weighted or unweighted data are reported.

### Analysis plan

As a first step in the analysis procedure, missing data were analysed and addressed. Overall, the missing rate was low, with an average of 3 % missing or invalid responses on all items in the survey (per item range: 0-18 %). However, a principled approach to data missingness is important even under relatively low missingness rates, especially if multivariate analyses including structural equation modelling (SEM) are planned. Therefore, missing observations were imputed under the Missingness At Random assumption using Amelia-2 [23] for R version 3.2.1 [24].

Next, the reliability of the scales was tested using maximum-likelihood factor analysis and Cronbach’s α coefficient for internal consistency. Scoring of contra-indicative items was reversed. Variables that were poor factor indicators (loadings <0.4) on a one-factor solution were excluded from the scales. After Cronbach’s α reliability coefficients were calculated, a SEM was constructed with the TPB constructs (attitudes, subjective norms, PBC and intention), past behaviour and current smoking behaviour as latent variables. The a priori hypothesis was that the full model comprising the five latent variables associated with intention would optimally fit the data. In optimisation steps, alternative models, consisting of a subset of the five initial latent variables were created and tested for their association with intention. Therefore, the SEM approach can be described as model-generating, starting with theory-based constructs.

The outcome variable (intention) is categorical. Therefore, diagonally weighted least squares (DWLS) with robust standard errors and mean and variance adjusted test statistics were used for the estimation of the SEM. SEM analyses were performed based on the covariance matrices using the R package *lavaan* version 0.5-19 [25]. The residual variances and the variances of exogenous latent variables are included in the model and set free. The metric of each latent variable is determined by fixing their variances to 1.0 (which gave the same results as fixing the first indicator to 1.0). The means of the observed variables are entered in the model.

To estimate SEMs with categorical outcomes and a DWLS estimator while taking survey weights in account is not possible in *lavaan* version 0.5-19. Therefore, a parametric bootstrapping procedure was performed in which the process of SEM estimation was repeatedly (1000 iterations) performed on a parametrically bootstrapped dataset, in which the probability for a given case to be sampled in the bootstrapped dataset was proportional to its survey weight. Through this approach, the application of survey weights in the SEM estimation process was computationally approached.

The SEMs were evaluated based on common SEM fit indices: (1) chi-square test of model fit (*χ*2), (2) comparative fit index (CFI), (3) and root mean square error of approximation (RMSEA). For the presentation of the SEM analyses, we adhered to Hoyle and Isherwood’s recommended reporting standards [26] and take in account the reporting recommendations by Jackson and colleagues [27].

## Results

### Participants

In total, 770 staff members submitted the survey via the submit button on the last page. Of those, 170 were excluded. The majority (*n* = 132) of these 170 were excluded because they worked for other mental health care organizations than the three types we intended to include in this study. Others were excluded because filling out the survey took them an unreasonably short (<7 min) or long (>6 h) time (*n =* 7), because of inconsistencies in their demographic data which indicated invalid input (*n* = 19) or because they were multivariate outliers based on Mahalanobis distances over all variables (*n* = 12). Of the remaining 600 respondents, 94 were excluded as they did not regularly have treatment contact with patients. The result was a net sample of 506 participants who had direct contact with patients. This can be considered a sufficient sample size based on a common rule-of-thumb for sample sizes in SEM (minimum of 10 cases per parameter [28]), and on a recent simulation study (*n* > 460 for relatively complex models [29]).

Based on weighted data, the majority of the respondents were females (70.0 %). Respondents had an average age of 42.5 years (*SD* = 12.0). Seventy-five percent had at least a BSc educational background. The most common vocational background of the respondents was in nursing (38.2 %), followed by social work (15.6 %), psychology (8.0 %), medicine (6.1 %), or other vocational backgrounds (e.g. drama therapists, music therapists; 2.4 %). The other 29.7 % did not have a vocational background in mental health. Analysis of the unweighted data indicated a slight underrepresentation of females (62.7 % vs. 70.0 % after applying survey weights) and an overrepresentation of respondents with a social work background (23.2 % vs. 15.6 % after applying survey weights) in the sample–compared to the weighted data.

### Descriptive statistics of items and latent variables

**Fig. 1:**
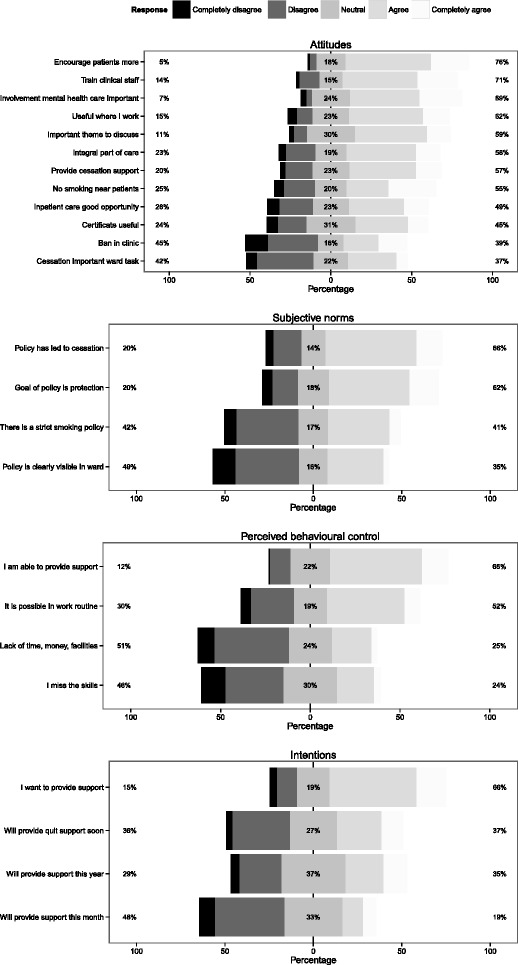
Measurement items for attitudes, subjective norms, perceived behavioural control and intention

The measurement items which comprise the latent variables attitudes (ATT), subjective norms (SN), perceived behavioural control (PBC) and intention (INT) are summarised in Fig. 1. The full list of items and response options for each latent variable is available as Additional file 1. The items used in the SEM analysis for the latent variables “past cessation support behaviour” (PB) and “smoking behaviour” (SMO) are listed in Table 1, with a summary of the responses.

**Table 1:** Responses to the items comprising the latent variables “Past cessation support behaviour” and “Smoking behaviour”

Latent variable	Item	Response
Past cessation support behaviour (PB)	Have you ever helped a patient quit?	No: 45.3 % (*n* = 229)
		Yes, not during the last year: 30.6 % (*n* = 155)
		Yes, during the last year: 24.1 % (*n* = 122)
	How many patients have you helped quit in the last year?	0 patients: 75.4 % (*n* = 381)
		1-3 patients: 16.5 % (*n* = 83)
		>3 patients: 8.3 % (*n* = 42)
	What percentage of your patients has received cessation support?	<10 % of patients: 73.7 % (*n* = 373)
		10 % - 30 % of patients: 15.7 % (*n* = 79)
		>30 % of patients: 10.6 % (*n* = 54)
Smoking behaviour (SMO)	Do you smoke cigarettes yourself?	I have never smoked: 29.3 % (*n* = 148)
		I used to smoke but quit: 42.1 % (*n* = 213)
		I am a regular smoker: 28.1 % (*n* = 142)
	How long after waking up do you smoke your first cigarette?	I don’t smoke daily: 80.2 % (*n* = 406)
		Within 5 min: 1.3 % (*n* = 7)
		Between 6 and 30 min: 8.7 % (*n* = 44)
		Between 31 and 60 min: 5.9 % (*n* = 30)
		Longer than 60 min: 3.8 % (*n* = 19)
	Do you intend to quit smoking?	I don’t smoke/not applicable: 71.6 % (*n* = 362)
		Within 6 months from now: 10.3 % (*n* = 52)
		Not within 6 months from now: 18.1 % (*n* = 92)

*Table note:* Percentages are based on weighted data. Counts are based on the *n* = 506 respondents with direct patient contact. Percentages for “Do you smoke cigarettes” do not count up to 100 % as 0.5 % of the respondents indicated “I don’t know” – those answers have been labelled as missing in the scale analyses

#### Attitudes

The scale has a good reliability (Cronbach’s α = 0.90; one factor with eigenvalue >1, based on unweighted data). In general, respondents held positive attitudes towards addressing smoking among patients. The majority of the respondents (76 %) indicated that patients should be encouraged more to quit smoking. Respondents were also supportive of providing more training to their direct colleagues to provide cessation support (71 %) and the involvement of mental health facilities in providing cessation support to patients (69 %). Respondents were notably less supportive of general smoking bans in mental health facilities (39 %) nor considered cessation support currently to be an important task in the ward they worked on.

#### Subjective norms

The scale has an acceptable reliability (α = 0.71; one factor with eigenvalue >1, based on unweighted data). The majority of the respondents thinks that smoking policies in their centre prescribe that patients should quit smoking (66 %), while a minority thinks the smoking policy in their centre is very prohibitive of smoking (41 %).

#### Perceived behavioural control

The scale’s reliability is acceptable but somewhat low (α = 0.65; one factor with eigenvalue >1, based on unweighted data). The majority of the respondents feel capable to provide cessation support (66 %).

#### Intention

The scale has a good reliability (α = 0.80; one factor with eigenvalue >1, based on unweighted data). Two thirds of the respondents wants to provide support, however only a minority (35 %) intends to actually do so over the next year.

#### Past behaviour

This scale showed an acceptable reliability (α = 0.71; one factor with eigenvalue >1, based on unweighted data). More than half of the respondents (55 %) indicates that he or she has never helped a patient quit smoking during his or her career. Only 8 % of the respondents has helped more than three patients quit smoking in the last year.

#### Respondent’s smoking behaviour

This scale has a good reliability of α = 0.82; one factor with eigenvalue >1, based on unweighted data). Twenty-eight percent of the respondents is a regular smoker, which is somewhat more than the average proportion of smokers in the general population in the Netherlands in 2014 (23 %) [30].

### Model fit using unweighted data

**Fig. 2:**
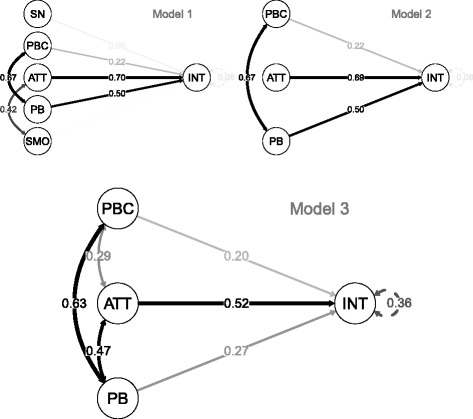
Initial Model 1, Model 2 with non-significant paths removed, and optimized Model 3. Figure note: SN: subjective norm, PBC: perceived behavioural control; ATT: behavioural attitude; PB: past behaviour (providing cessation support to patients); SMO: current smoking behaviour; INT: intention to provide cessation support to patients in the near future; Model 1: Initial model with all possible relevant constructs included; Model 2: Initial model after insignificant paths and constructs have been removed; Model 3: Final model based on model 2 with covariances specified based on modification indices; The numbers in the paths between two latent constructs are standardized parameter estimates. Covariance matrices for the three models are available as Additional file 2. Analyses are based on unweighted data

As a first step in the model fitting phase, a model including all latent variables possibly associated with the intention to provide (more) cessation support in the near future was estimated (model in the top left in Fig. 2). Model 1 showed suboptimal fit to the data, as indicated by the chi-square statistic (*χ*^2^ (529) = 4273, *p* < 0.001), and the RMSEA (0.109). The CFI (0.989) indicated the model fitted the data well, however some of the estimated variances were negative; another indication of model misfit. As can be partly observed from the standardized parameter estimates in Fig. 2, the regression coefficient estimates from subjective norm to intention, and from smoking behaviour to intention were found to be weak and non-significant (SN- > INT = 0.199, SE = 0.179, *p* = 0.267; SMO- > INT = −0.037, SE = 0.154, *p* = 0.812). These two latent variables were therefore removed in Model 2. This resulted in a lower chi-square statistic (*χ*^2^ (327) = 3498, *p* < 0.001), but did not improve the RMSEA (0.127) nor the CFI (0.936). For the third model presented in Fig. 2, covariances have been specified based on modification indices. Six covariances that led to an expected significant change in chi-square (with α = 0.01; power = 0.9; delta ≥ 0.2) were added to the model: three between PB and PBC, PB and ATT, PBC and ATT and three and three covariances between pairs of items. These were the items “Important theme to discuss” and “Involvement mental health care important”; “No smoking near patients” and “Ban in clinic” and the PBC items “It is possible in work routine” and “I miss the skills”. Compared to Model 1 and 2, Model 3 showed a better fit to the data: *χ*^2^ (322) = 790, *p* < 0.001); RMSEA = 0.049; CFI = 0.991. All three models were identified. In none of the models, any samples were lost due to non-convergence or other analysis problems.

### Model fit using weighted data

**Fig. 3:**
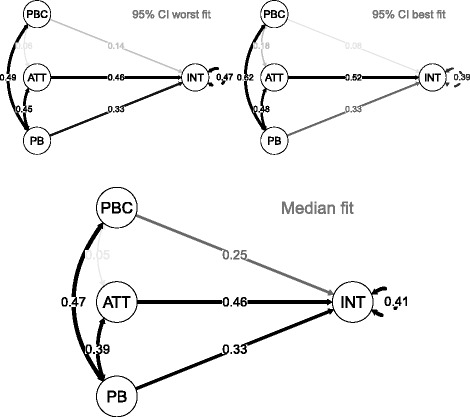
Median model, 95 % CI worst fitting, and 95 % CI best fitting model. Figure note: PBC: perceived behavioural control; ATT: behavioural attitude; PB: past behaviour (providing cessation support to patients); INT: intention to provide cessation support to patients in the near future; Model ‘Median fit’: Final optimized model identified using bootstrapped data (1000 iterations) using the median fitting data (based on *χ* ^2^): 50 % of the bootstrapped datasets fitted the model better, 50 % fitted the model worse; Model ‘95 % CI worst fit’: 97.5 % of the bootstrapped datasets fit the model better, 2.5 % fit the model worse (based on *χ* ^2^); Model ‘95 % CI best fit’: 2.5 % of the bootstrapped datasets fit the model better, 97.5 % fit the model worse (based on *χ* ^2^); The numbers in the paths between two latent constructs represent standardized parameter estimates, Covariance matrices for the three presented fits are available as Additional file 2

As it was not directly possible to include the survey weights in the SEM analysis using the DWLS estimator, we chose to test the sensitivity of the model fitting results to the weighted characteristics of the data. The optimized model (Model 3) fitted originally to the unweighted data was fitted iteratively (1000 times) to the bootstrapped datasets, to compare the median fit - with 95 % confidence intervals (CIs) under the weighted bootstrapping approach to the result under the unweighted approach. Figure 3 presents the model with standardized parameter estimates from the median fitting dataset, and the two models fitted using the upper and lower 95 % CI datasets (fit was evaluated using the chi-square statistic).

The fit indices for the bootstrapped data with median model fit were *χ*^2^ (322) = 1188, *p* < 0.001); RMSEA = 0.067; CFI = 0.983; the ‘95 % CI worst fit’ indices were *χ*^2^ (322) = 1518, *p* < 0.001); RMSEA = 0.079; CFI = 0.975; and the ‘95 % CI best fit’ indices were *χ*^2^ (322) = 953, *p* < 0.001); RMSEA = 0.057; CFI = 0.988. The median model replicated the pattern and strength of the associations observed when unweighted data were used.

## Discussion

The findings of this study indicated that in general, mental health staff in the Netherlands support encouraging patients more to quit smoking. The majority of the staff members feels capable to provide cessation support if needed, however only a minority of them intends to actually provide support over the next year. More than half of them have no experience in helping a patient quit smoking.

Theoretically derived constructs associated with intentions to provide smoking cessation support to patients were identified. Attitudes towards providing cessation support, perceived behaviour control and past experience in providing support were strongly associated with the intention to provide future support. For subjective norms toward smoking (cessation) for patients and respondents own smoking behaviour we found limited evidence of an association with intention.

The limited association between subjective norms and intention is in line with previous findings. In the meta-analysis by Armitage and Conner [15] it is reported that subjective norm is more weakly correlated with intention than attitude and perceived behavioural control. A number of possible explanations for this limited correlation have been suggested. Some argue that the lack of association between the two indicates that intentions are influenced primarily by intra-personal factors and not as much by what others are perceived to think or do [14, 31]. Another explanation is in the distinction between injunctive norms (i.e. what significant others think the person ought to do) and descriptive norms (i.e. what significant others themselves do). The subjective norm component of the TPB is an injunctive social norm (perceived social pressure, in this case: perceived strictness of smoking policies), while the results of a meta-analysis based upon 14 TPB studies involving a total sample size of *N* = 5810, covering a wide range of behavioural domains, provides strong evidence in support of the predictive validity of descriptive norms, over injunctive norms [31]. In addition, the inconsistent findings in the literature regarding the impact of strict smoking policies on smoking prevalence among patients and staff of mental health institutes [32–34] are also in line with the theoretically derived finding that there is only a weak link between subjective norms/policy regarding smoking cessation support and the intention to provide cessation support.

The absence of a direct association between staff smoking behaviour and their intentions to provide cessation support has some precedents in the literature, although findings are mixed. A large cross-sectional survey of 3482 nurses working in 35 hospitals in the USA, did not find differences between smoking and non-smoking nurses in the likelihood that nurses asked patients about smoking, gave cessation advice, assessed willingness to quit, assisted in quitting or recommended medications/referred to a quit line [13]. In a recent study performed in Czech Republic, the same author found mixed results [35]. In a study by Slater and colleagues [36], 1074 smoking nurses rated the need for and potential of the nurse’s role in patients’ smoking cessation lower than non-smokers and ex-smokers. However, smoking and ex-smoking nurses rated their responsibility to help patients who wanted to quit higher than non-smokers.

### Limitations

The reported findings in this study and its implications should be interpreted in the light of the limitations. A first limitation of this study is that the survey is cross-sectional in nature, thereby hampering the possibility to (longitudinally) model the impact of the evaluated constructs on actual behaviour (i.e. provision of smoking cessation support). Based on a meta-analysis that included 47 experimental tests of intention–behaviour relations, it is known that a medium-to-large change in intention (d = 0.66) leads only to a small-to-medium change in behaviour (d = 0.36) [37]. Thus, intention has a significant impact on behaviour, but the size of this effect is considerably small.

A second limitation of this study is that the sample is comprised of self-referred respondents from mental health institutes. Therefore, the representativeness of the sample is a matter for debate. An assumption underlying the presentation of the results as potentially generalizable to the wider population of mental health care providers is that the associations between variables in this study would also have been found in a representative sample of mental health workers. Survey weights were calculated in order to optimize the representativeness of our sample regarding type of organization the participant worked for, number of inhabitants of the province where the professional worked, gender, age, part time factor and type of function.

A third limitation is that although the fit of Model 3 and the bootstrapped model (Median) was acceptable or nearly acceptable according to common cut-off points for CFI (≥.95) [38] and RMSEA (<.07) [39], there still was considerable misfit of the model to the data, as evidenced by the chi-square statistic. In addition, in order to construct Model 3 from Model 2, modification indices were used to identify data-driven model optimisations in the form of the inclusion of six covariance paths to the model. Moreover, although the covariances between the latent constructs and measurement items have face validity, it should be acknowledged that post hoc modifications to models, for example based on modification indices, should be done sparingly and only when the modifications are plausible [27, 40].

### Implications

For many years the mental health treatment community tolerated or even encouraged smoking [4]. To date, mental health professionals and treatment organisations respond differently to this challenge. Although progress has been made in recent years, many (45.3 % in the current sample) mental health workers have never addressed their patients’ smoking behaviour. This study has some implications for future interventions to further promote these cessation support activities among mental health staff. Based on our results, it is best to address staff attitudes towards providing support, and to increase their perceived behavioural control towards supporting patients to quit smoking. The third identified correlate of intention, past cessation support behaviour, cannot directly be influenced. It should be acknowledged that changes in clinicians’ behaviour tend not to happen overnight [41]. There is however some evidence that an implementation strategy to support mental health professionals in providing smoking cessation support should focus on changing attitudes and perceived behavioural control, based on our and previous [20, 21] findings. Based on findings and frameworks developed in the implementation science discipline, features such as including a focus on engaging stakeholders and iterative Deming cycles (“plan-do-check-act”) in addition to understanding and targeting determinants of behaviour are key to bring about change in professionals’ behaviour [41].

## Conclusion

This study demonstrated that attitudes, perceived behavioural control and past behaviour of mental health workers are the strongest correlates of intention toward providing smoking cessation support to patients among the five theory-derived constructs tested in a SEM approach. Subjective norms and the mental health workers’ smoking behaviour were found to be notably less strong correlates of intention. These findings are to a great extent in line with previous findings and underline inconsistencies in the literature regarding the association between health workers’ smoking behaviour and their intentions to help patients stop smoking. Based on our findings, an implementation strategy to provide mental health care patients with smoking cessation support should best target staff attitudes and perceived behavioural control.

## Additional files

Additional file 1: List of original survey items and response options. (XLSX 17 kb) Additional file 2: Covariance matrices of the fitted structural equation models. (XLSX 50 kb)

## Abbreviations

ATT: Attitudes
BSc: Bachelor of science
CFI: Comparative fit index
CI: Confidence interval
DWLS: Diagonally weighted least squares
INT: Intention
PB: Past cessation support behaviour
PBC: Perceived behavioural control
RMSEA: Root mean square error of approximation
SD: Standard deviation
SEM: Structural equation modelling
SMO: Smoking behaviour
SN: Subjective norms
TPB: Theory of planned behaviour
